# Discovery of Potential Therapeutic Drugs for COVID-19 Through Logistic Matrix Factorization With Kernel Diffusion

**DOI:** 10.3389/fmicb.2022.740382

**Published:** 2022-02-28

**Authors:** Xiongfei Tian, Ling Shen, Pengfei Gao, Li Huang, Guangyi Liu, Liqian Zhou, Lihong Peng

**Affiliations:** ^1^School of Computer Science, Hunan University of Technology, Zhuzhou, China; ^2^College of Life Sciences and Chemistry, Hunan University of Technology, Zhuzhou, China; ^3^Academy of Arts and Design, Tsinghua University, Beijing, China; ^4^The Future Laboratory, Tsinghua University, Beijing, China

**Keywords:** anti-SARS-CoV-2 drug, virus-drug association, logistic matrix factorization, kernel diffusion, molecular docking

## Abstract

Coronavirus disease 2019 (COVID-19) is rapidly spreading. Researchers around the world are dedicated to finding the treatment clues for COVID-19. Drug repositioning, as a rapid and cost-effective way for finding therapeutic options from available FDA-approved drugs, has been applied to drug discovery for COVID-19. In this study, we develop a novel drug repositioning method (VDA-KLMF) to prioritize possible anti-SARS-CoV-2 drugs integrating virus sequences, drug chemical structures, known Virus-Drug Associations, and Logistic Matrix Factorization with Kernel diffusion. First, Gaussian kernels of viruses and drugs are built based on known VDAs and nearest neighbors. Second, sequence similarity kernel of viruses and chemical structure similarity kernel of drugs are constructed based on biological features and an identity matrix. Third, Gaussian kernel and similarity kernel are diffused. Forth, a logistic matrix factorization model with kernel diffusion is proposed to identify potential anti-SARS-CoV-2 drugs. Finally, molecular dockings between the inferred antiviral drugs and the junction of SARS-CoV-2 spike protein-ACE2 interface are implemented to investigate the binding abilities between them. VDA-KLMF is compared with two state-of-the-art VDA prediction models (VDA-KATZ and VDA-RWR) and three classical association prediction methods (NGRHMDA, LRLSHMDA, and NRLMF) based on 5-fold cross validations on viruses, drugs, and VDAs on three datasets. It obtains the best recalls, AUCs, and AUPRs, significantly outperforming other five methods under the three different cross validations. We observe that four chemical agents coming together on any two datasets, that is, remdesivir, ribavirin, nitazoxanide, and emetine, may be the clues of treatment for COVID-19. The docking results suggest that the key residues K353 and G496 may affect the binding energies and dynamics between the inferred anti-SARS-CoV-2 chemical agents and the junction of the spike protein-ACE2 interface. Integrating various biological data, Gaussian kernel, similarity kernel, and logistic matrix factorization with kernel diffusion, this work demonstrates that a few chemical agents may assist in drug discovery for COVID-19.

## Introduction

A novel coronavirus disease named COVID-19, caused by coronavirus SARS-CoV-2, is spreading around the globe. As of 3 December 2021, more than 263 million confirmed cases of SARS-CoV-2 infection and 5,232 thousand confirmed cases of SARS-CoV-2-caused death have been reported (WHO, 2021). The rapid transmission of SARS-CoV-2 has become a severe threat to public health worldwide (Baker et al., 2020; Hopman et al., 2020; Khan M. T. et al., 2021). Although its vaccines have been studied (Li et al., 2021), it is an immediate urgency to find promising antiviral drugs for COVID-19 therapies (Mahdian et al., 2020; Saxena, 2020).

However, under such an urgent situation, it is almost impossible to research and develop a new drug for patients with the COVID-19 infections since designing a new drug may spend more than 10 years (Liu et al., 2020; Yang et al., 2020). It might be an effective alternative to find possible therapeutic clues from Food and Drug Administration (FDA)-approved drugs, that is, drug repurposing (Liu et al., 2016; Yang et al., 2016; Chu et al., 2020; Masoudi-Sobhanzadeh, 2020; Zhang et al., 2020, 2021). Now, researchers worldwide have focused on repositioning the FDA-approved drugs for COVID-19. Since these drugs have been tested for the efficacy, safety, and toxicity in the clinical trials, they can be fast applied as clinically available drugs against COVID-19 (Wu et al., 2020). Multiple examples of repositioned drugs, such as antiviral drugs and host-targeting treatment, are or have been clinical trials for COVID-19 (Tang et al., 2020). Computational methods for identifying potential options against COVID-19 can be categorized into structure-based virtual screening methods (Khan M. T. et al., 2021) and network-based methods (Dotolo et al., 2020).

To capture possible antiviral drugs against SARS-CoV-2, a vast amount of structure-based virtual screening methods are carried out. The type of methods uses molecular docking and dynamics simulation techniques to measure binding capabilities between potential anti-COVID-19 drugs and targets. For example, Elfiky (2020) and Muralidharan et al. (2020) combined molecular docking and molecular dynamics simulation. Islam et al. (2021) integrated docking with two approaches, molecular dynamics simulation, and *in silico* absorption, distribution, metabolism, excretion, and toxicity (ADMET) profile. Kandeel et al. (2020) applied molecular docking, molecular dynamics simulation of top 10 hits, and free energy calculation. Khan et al. (2020a) designed an integrated computational framework for key residue identification via an alanine scanning strategy and an extensive simulation, a cryo-EM structure for novel drug identification based on computational virtual screening and molecular docking (Khan et al., 2020b), a multi-step drug screening method to shortlist potential drugs (Khan S. et al., 2021), and a structural and biomolecular simulation technique for revealing the impact of specific mutations in the B.1.617 variant (Khan A. et al., 2021). Wang et al. (2020) detected inhibition affect of human defensin-5 against SARS-CoV-2 invasion combining molecular dynamics simulation and statistical analysis. Elmezayen et al. (2020) used molecular docking for top-ranked compounds, molecular dynamics simulations, ADMET profile prediction, and free energy computation. Wang C. et al. (2021) found a versatile antimicrobial peptide that can be used as an inhibitor of SARS-CoV-2 attachment based on dual mechanisms.

Network-based methodologies are widely applied to drug repositioning by integrating multiple data sources. In these methods, nodes denote drugs, diseases, or targets, while edges denote interactions or associations between nodes. Network-based methods contain network-based clustering methods and network-based propagation methods (Messina et al., 2020; Sadegh et al., 2020). Network-based clustering methods have been developed to find novel drug-target interactions or drug-disease associations by finding biological modules (for example, drug-target, drug-disease, drug-drug) using clustering algorithms. Network-based propagation methods used network proximity and network propagation algorithms to model associations between drugs, targets, and COVID-19-related diseases. For example, Peng et al. (2020) and Zhou et al. (2020) separately used bipartite local model and the KATZ measurement to find potentially suitable drugs against COVID-19 and validated the predicted results by molecular docking and recent publications. Meng et al. (2021) proposed a similarity constrained probabilistic matrix factorization method to find new Virus-Drug Associations (VDAs). Fiscon et al. (2021) developed a searching off-label drug and network method to uncover interactions between targets and disease-specific proteins. Based on the above studies, (Peng et al., 2021) developed a random walk with restart-based VDA prediction model to discover possible anti-SARS-CoV-2 drugs on the constructed three VDA datasets. These methods effectively discovered possible antiviral drugs for the treatment of COVID-19.

In this study, we develop a novel VDA prediction method, VDA-KLMF, to find potential chemical agents for COVID-19. VDA-KLMF integrates virus sequences, drug chemical structures, known VDAs, Gaussian kernel, similarity kernel, and Logistic Matrix Factorization with Kernel diffusion. VDA-KLMF is compared with two state-of-the-art VDA prediction models [VDA-KATZ (Zhou et al., 2020) and VDA-RWR (Peng et al., 2021)] and three classical association identification models [NGRHMDA (Huang et al., 2017), LRLSHMDA (Wang et al., 2017), and NRLMF (Liu et al., 2016)] based on fivefold cross validations on viruses, drugs, and VDAs on three VDA datasets. Experimental results show that VDA-KLMF computes the optimal recalls, AUCs, and AUPRs, significantly improving VDA identification performance. Four chemical agents (remdesivir, ribavirin, nitazoxanide, and emetine) coming together on any two VDA datasets are inferred to be underlying anti-COVID-19 drugs.

Molecular docking is an important drug discovery tool applied to find the best appropriate intermolecular binding between a chemical agent and a target or two proteins. It can effectively elucidate fundamental biochemical processes and characterize activity of ligands binding target proteins (McConkey et al., 2002). In this manuscript, a molecule docking software, AutoDock (Morris et al., 2009), is used to measure the molecular activities of the predicted four antiviral small molecules at the junction of the SARS-Cov-2 Spike (S) protein-angiotensin-converting enzyme 2 (ACE2) interface. The dockings show that the four drugs have higher binding energies with two key residues (K353 and G496).

## Materials and Methods

### Materials

Three VDA datasets were provided by Peng et al. (2021). Dataset 1 contains 96 VDAs from 11 viruses and 78 drugs. Dataset 2 contains 770 VDAs from 69 viruses to 128 drugs. Dataset 3 contains 407 VDAs from 34 viruses and 203 drugs. The virus sequences and drug chemical structures can be downloaded from the NCBI (Sayers et al., 2021) and DrugBank (Wishart et al., 2018) databases, respectively. Virus sequence similarity matrix ***S***_*v*_ and drug chemical structure similarity matrix ***S***_*d*_ can be computed by MAFFT (Katoh et al., 2019) and RDKit (Landrum, 2021), respectively. The details are shown in Table 1.

**TABLE 1:** Statistics for three VDA networks.

Datasets	Viruses	Drugs	VDAs
Dataset 1	12	78	96
Dataset 2	69	128	770
Dataset 3	34	203	407

All virus-drug pairs in a dataset can be characterized as a matrix ***Y***:


(1) $$Y_{ij}=\left\{ \begin{array}{cc} 1 & \text{if}v_{i}\text{associates}\text{with}d_{j}\\ 0 & \text{otherwise} \end{array}\right.$$

where *v_i_* and *d_j_* represent the *i*th virus and *j*th drug, respectively.

### Problem Formalization

**FIGURE 1:**
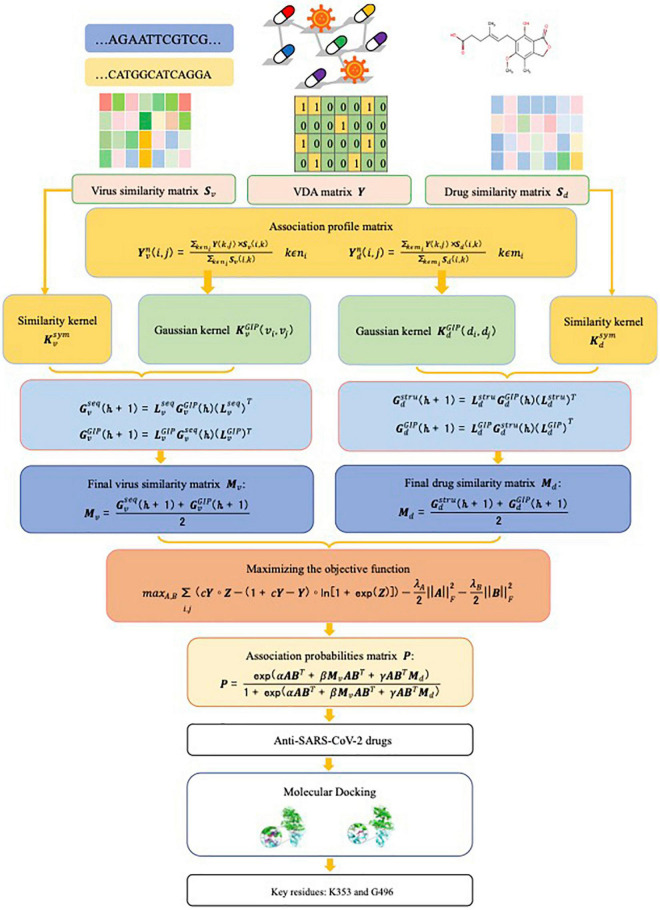
The flowchart of the VDA-KLMF framework.

Given virus similarity matrix ***S***_*v*_, drug similarity matrix ***S***_*d*_, and VDA matrix ***Y***, our task is to quantify the interplays between viruses and drugs, which can be divided into four scenarios: (1) known virus-known drug association, that is, a virus associates with no less than one drug and a drug associates with no less than one virus; (2) known virus-new drug association, that is, a virus interacts with at least one drug and a new drug does not interact with any virus; (3) new virus-known drug association, that is, a new virus does not associate with any drug and a drug interacts with at least one virus; (4) new virus-new drug association, that is, both virus and drug have no any association information. Our goal is to exploit a novel model to boost the VDA prediction performance. In particular, the model assigns an association probability to a virus-drug pair to measure the likelihood of interplay between the virus and the drug. The higher the probability is, the more likely the virus and the drug are associated with each other. Figure 1 illustrates the flowchart of the VDA-KLMF model.

### Gaussian Kernel Construction

SARS-CoV-2 is a new single strand RNA virus and has no any associated drug. That is, there may exist the scenario of new virus (for example, SARS-CoV-2) and new drug when a VDA dataset is split during cross validations. The nearest neighbor information of a virus/drug contributes to prioritizing VDAs related to the virus/drug. To find interacting drugs for a virus *v_i_*, its Gaussian kernel is constructed as follows.

First, its association profile is computed based on its nearest neighbor information by Eq. (2):


(2) $${\text{Y}}_{v}^{a}\left(i,j\right)=\sum_{k\epsilon n_{i}}\text{Y}\left(k,j\right)\times {\text{S}}_{v}\left(i,k\right)k\epsilon n_{i}$$

where *n_i_* represents nearest neighbors of *v_i_*, and *k* is a hyper-parameter and denotes the number of nearest neighbors of *v_i_*.

Second, the computed association profile is normalized by Eq. (3):


(3) $${\text{Y}}_{v}^{n}\left(i,j\right)=\frac{{\text{Y}}_{v}^{a}\left(i,j\right)}{\sum_{k\epsilon n_{i}}{\text{S}}_{v}\left(i,k\right)}k\epsilon n_{i}$$

Finally, Gaussian kernel ${\text{K}}_{v}^{GIP}$ of viruses is calculated via the normalized association profiles by Eq. (4):


(4) $${\text{K}}_{v}^{GIP}\left(v_{i},v_{j}\right)=\exp\left(-\frac{{||{\text{Y}}_{v}^{n}\left(i,k\right)-{\text{Y}}_{v}^{n}\left(i,j\right)||}^{2}}{\sigma }\right)$$

where *σ* is the kernel bandwidth. Similarly, Gaussian kernel ${\text{K}}_{d}^{GIP}$ of drugs can be computed.

### Similarity Kernel Construction

Sequence information of viruses and chemical structure information of drugs help VDA candidate screening. To comprehensively consider these data, original two similarity matrices are transformed into two kernel matrices (${\text{K}}_{v}^{sym}$ and ${\text{K}}_{d}^{sym}$). First, original virus similarity matrix is converted to a symmetric matrix ${\text{S}}_{v}^{sym}$ by Eq. (5):


(5) $${\text{S}}_{v}^{sym}=\frac{1}{2}\left({\text{S}}_{v}+{\text{S}}_{v}^{T}\right)$$

Then, the symmetrized matrix ${\text{S}}_{v}^{sym}$ is transformed into a positive semi-definite matrix ${\text{K}}_{v}^{sym}$ by Eq. (6):


(6) $${\text{K}}_{v}^{sym}={\text{S}}_{v}^{sym}+\varepsilon I$$

where ***I*** is an identity matrix, and *ε* is a parameter. Similarly, ${\text{K}}_{d}^{sym}$ can be calculated.

### Similarity Diffusion

For a virus, its Gaussian kernel only depicts the similarities between the virus and its *k* nearest neighbors, the remaining information is discarded. To characterize virus features, inspired by a kernel technique proposed by Hao et al. (2016), we diffused two different types of virus similarity into a final kernel matrix.

First, the local virus similarity matrices are built based on Gaussian kernel and similarity kernel by Eqs (7) and (8), respectively:


(7) $${\text{L}}_{v}^{GIP}\left(i,j\right)=\{\begin{array}{c} \begin{array}{cc} \frac{K_{v}^{GIP}\left(i,j\right)}{\sum_{k\epsilon n_{i}}K_{v}^{GIP}\left(i,k\right)} & j\epsilon n_{i} \end{array}\\ \begin{array}{cc} 0 & \mathrm{otherw}\text{ise} \end{array} \end{array}$$

(8) $${\text{L}}_{v}^{seq}\left(i,j\right)=\{\begin{array}{c} \begin{array}{cc} \frac{K_{v}^{sym}\left(i,j\right)}{\sum_{k\epsilon n_{i}}K_{v}^{sym}\left(i,k\right)} & j\epsilon n_{i} \end{array}\\ \begin{array}{cc} 0 & \mathrm{otherw}\text{ise} \end{array} \end{array}$$

Second, the global virus similarity matrices ${\text{G}}_{v}^{GIP}$ and ${\text{G}}_{v}^{seq}$are produced by iteratively updating by Eqs. (9) and (10).


(9) $${\text{G}}_{v}^{GIP}\left(h+1\right)={\text{L}}_{v}^{GIP}{\text{G}}_{v}^{seq}\left(h\right){\left({\text{L}}_{v}^{GIP}\right)}^{T}$$

(10) $${\text{G}}_{v}^{seq}\left(h+1\right)={\text{L}}_{v}^{seq}{\text{G}}_{v}^{GIP}\left(h\right){\left({\text{L}}_{v}^{seq}\right)}^{T}$$

where ${\text{G}}_{v}^{GIP}\left(h+1\right)$ and ${\text{G}}_{v}^{seq}\left(h+1\right)$ represent global Gaussian kernel and similarity kernel matrices generated at *h*-th iteration, respectively. And ${\text{G}}_{v}^{GIP}\left(1\right)={\text{K}}_{v}^{sym}$ and ${\text{G}}_{v}^{seq}\left(1\right)={\text{K}}_{v}^{GIP}$.

Finally, virus similarity matrix ***M***_*v*_ is integrated by Eq. (11):


(11) $${\text{M}}_{v}=\frac{1}{2}\left({\text{G}}_{v}^{GIP}\left(h+1\right)+{\text{G}}_{v}^{seq}\left(h+1\right)\right)$$

Similarly, drug similarity matrix ***M***_*d*_ can be computed.

### Methods

After the diffused virus similarity matrix ***M***_*v*_ and drug similarity matrix ***M***_*d*_ are computed, a Logistic Matrix Factorization model (VDA-KLMF) with Kernel diffusion (Liu et al., 2020) is then designed for VDA discovery. Viruses and drugs are first randomly mapped into two latent vector spaces ***A***ϵ*R^m×r^* and ***B***ϵ*R^n×r^* with the dimension of *r*. And association probability for each virus-drug pair can be calculated by Eq. (12):


(12) $$\text{P}=\frac{\exp\left(\alpha {\text{AB}}^{T}+\beta {\text{M}}_{v}{\text{AB}}^{T}+\gamma {\text{AB}}^{T}{\text{M}}_{d}\right)}{1+\exp\left(\alpha {\text{AB}}^{T}+\beta {\text{M}}_{v}{\text{AB}}^{T}+\gamma {\text{AB}}^{T}{\text{M}}_{d}\right)}$$

where α, β, and γ are smoothing coefficients with the summation of 1, ***B***^*T*^ denotes the transpose of ***B***. Inspired by the method provided by Liu et al. (2016), under the assumption that all samples are independent, interplays between viruses and drugs can be rewritten by assigning each known VDA as a confident value of *c* by Eq. (13):


(13) $$p\left(\text{Y}|\text{A},\text{B}\right)=\prod_{i=1}^{m}\prod_{j=1}^{n}P_{ij}^{cY_{ij}}{\left(1-P_{ij}\right)}^{1-Y_{ij}}$$

where *P*_*ij*_ denotes association probability between the *i*-th virus and the *j*-th drug. Known VDAs are validated by wet experiments and more reliable, therefore, *c* is assigned as a higher value. Assume that the two vectors follow the zero-mean spherical Gaussian distribution defined by Eqs. (14) and (15):


(14) $$p\left(\text{A}|\sigma_{v}^{2}\right)=\prod_{i=1}^{m}N\left(a_{i}|0,\sigma_{v}^{2}\text{I}\right)$$

(15) $$p\left(\text{B}|\sigma_{d}^{2}\right)=\prod_{j=1}^{n}N\left(b_{j}|0,\sigma_{d}^{2}\text{I}\right)$$

where $\sigma_{v}^{2}$ and $\sigma_{d}^{2}$ are two parameters used to control the variances of Gaussian distribution, *a_i_* and *b_j_* refer to potential variables for the *i*-th virus and the *j*-th drug, respectively. ***I*** is an identity matrix. We can obtain the following distribution based on the Bayesian inference by Eq. (16):


(16) $$p\left(\text{A},\text{B}|\text{Y},\sigma_{v}^{2},\sigma_{d}^{2}\right)\propto p\left(\text{Y}|\text{A},\text{B}\right)p\left(\text{A}|\sigma_{v}^{2}\right)p\left(\text{B}|\sigma_{d}^{2}\right)$$

The log formula of the posterior distribution can be represented as Eq. (17):


(17) $$\begin{array}{c} lnp\left(\text{A},\text{B}|\text{Y},\sigma_{v}^{2},\sigma_{d}^{2}\right)\\ =\sum_{i,j}(c\text{Y}ο\left(\alpha {\text{AB}}^{T}+\beta {\text{M}}_{V}{\text{AB}}^{T}+\gamma {\text{AB}}^{T}{\text{M}}_{D}\right)\\ -\left(1+\text{c}\text{Y}-\text{Y}\right)ο\ln\left[1+\text{exp}\left(\alpha {\text{AB}}^{T}+\beta {\text{M}}_{V}{\text{AB}}^{T}+\gamma {\text{AB}}^{T}{\text{M}}_{D}\right)\right])\\ -\frac{\lambda_{A}}{2}\sum_{i=1}^{m}{||{\text{a}}_{i}||}_{2}^{2}-\frac{\lambda_{B}}{2}\sum_{j=1}^{n}{||{\text{b}}_{j}||}_{2}^{2}+C \end{array}$$

where $\lambda_{A}=\frac{1}{\sigma_{v}^{2}}$, $\lambda_{B}=\frac{1}{\sigma_{d}^{2}}$, $||\cdot |{|}_{2}^{2}$ represents the spectral norm, and ^ο^ denotes the Hadamard product. Thus the latent variable virus matrix ***A*** and drug matrix ***B*** can be generated by maximizing an objective function defined by Eq. (18):


(18) $$\begin{array}{c} max_{A,B}\sum_{i,j}(c\text{Y}ο\left(\alpha {\text{AB}}^{T}+\beta {\text{M}}_{V}{\text{AB}}^{T}+\gamma {\text{AB}}^{T}{\text{M}}_{D}\right)-\\ \left(1+c\text{Y}-\text{Y}\right)ο\text{ln}\left[1+\exp\left(\left(\alpha {\text{AB}}^{T}+\beta {\text{M}}_{V}{\text{AB}}^{T}+\gamma {\text{AB}}^{T}{\text{M}}_{D}\right)\right)\right]\\ -\frac{\lambda_{A}}{2}{||A||}_{F}^{2}-\frac{\lambda_{B}}{2}{||B||}_{F}^{2} \end{array}$$

where $||\cdot |{|}_{F}^{2}$ represents the Frobenius norm.

According to the work provided by Liu et al. (2016), ***A*** and ***B*** can be solved by Eqs. (19) and (20):


(19) $$\begin{array}{c} \frac{\partial LL}{\partial A}=c\left(\alpha \text{I}+\beta {\text{M}}_{v}^{T}\right)\text{Y}B\\ +\gamma \left(c\text{Y}-\text{R}\right){\text{M}}_{d}^{T}\text{B}-\left(\alpha \text{I}+\beta {\text{M}}_{v}^{T}\right)\text{RB}-\lambda_{A}\text{A} \end{array}$$

(20) $$\begin{array}{c} \frac{\partial LL}{\partial B}=c\left(\alpha \text{I}+\gamma {\text{M}}_{d}\right){\text{Y}}^{T}\text{A}\\ +\beta \left(c{\text{Y}}^{T}-{\text{R}}^{T}\right){\text{M}}_{v}\text{A}-\left(\alpha \text{I}+\gamma {\text{M}}_{d}\right){\text{R}}^{T}\text{A}-\lambda_{A}\text{B} \end{array}$$

where $\text{R}=\left(1+c\text{Y}-\text{Y}\right)ο\frac{1}{1+\text{exp}\left(-\left(\alpha {\mathrm{AB}}^{T}+\beta M_{v}{\mathrm{AB}}^{T}+\gamma {\mathrm{AB}}^{T}M_{d}\right)\right)}$, ***A*** and ***B*** can be updated based on the AdaGrad algorithm (Duchi et al., 2011).

### Molecular Docking

Molecular docking is utilized to measure dynamics and binding energies between the predicted antiviral compounds against SARS-CoV-2 and the junction of the S protein-ACE2 interface. Similar to the molecular docking process provided by Peng et al. (2021), we first downloaded structures of the S protein and ACE2 and chemical structures of drugs from the RCSB Protein Data Bank (Rose et al., 2016) and the DrugBank databases (Wishart et al., 2018), respectively. Second, solvent and organic compounds were removed and the receptor proteins were preprocessed based on PyMOL (Schrodinger, 2010). Third, atoms from receptors were set to the AD4 type. Finally, AutoDock was applied to implement molecular docking. During docking, the predicted anti-COVID-19 drugs was used as ligands and the junction of the S protein-ACE2 interface was taken as receptor. Binding pocket was set via AutoGrid4, the grid size was 126 × 126 × 126, and Lamarckian genetic algorithm was selected as the search method. The detailed processes were set the same as ones provided by Peng et al. (2021).

## Results

### Experimental Settings and Evaluation Metrics

We perform experiments to evaluate the performance of the VDA-KLMF method. Given a VDA matrix ***Y***_*n*×*m*_ between *n* viruses and *m* drugs, inspired by Cross Validation (CV) provided by Peng et al. (Peng et al., 2021), three different 5-fold CVs, CV on viruses (CV1), CV on drugs (CV2), and CV on virus-drug pairs (CV3), are implemented. Under CV1, in each round, 80% viruses are used to train VDA prediction models and the remaining 20% of viruses are used to test the performance of these models. Under CV2, in each round, 80% drugs are used to train VDA prediction models and the remaining 20% of drugs are used to test their performance. Under CV3, in each round, 80% VDAs are used to train VDA prediction models and the remaining 20% of VDAs are used to test their performance. The three CVs correspond to VDA prediction for a new virus, a new drug, or based on known VDA data.

The number of iterations *h* is set as 100. The confident level *c* of known VDA, the number of neighbors *k*, weights λ_*A*_, and λ_*B*_ are set in the range of [3, 10], [1, 10], [1, 10], and [1, 10], respectively. We repeatedly implemented experiments for 100 times and used random search approach to select the optimal parameters. The optimal parameter combinations of VDA-KLMF and other five VDA prediction methods (NGRHMDA, LRLSHMDA, NRLMF, VDA-KATZ, and VDA-RWR) are shown in Table 2.

**TABLE 2:** The optimal parameter combinations of six VDA prediction methods.

Method	Dataset 1	Dataset 2	Dataset 3
NGRHMDA	α = 0.4, β = 0.8	α = 0.6, β = 0.9	α = 0.9, β = 0.9
LRLSHMDA	μ*M* = 0.9, μ*D* = 0.3	μ*M* = 0.8, μ*D* = 0.1	μ*M* = 0.6, μ*D* = 0.1
NRLMF	*r* = 8, β = 1, α = 4, θ = 0.5, λ_*t*_ = λ_*d*_ = 0.03125	*r* = 5, β = 1, α = 4, θ=0.125, λ_*t*_ = λ_*d*_ = 2	*r* = 10, β = 1, α = 4, θ = 0.25, λ_*t*_ = λ_*d*_ = 2
VDA-KATZ	β = 0.04, *w*_1_ = *w*_2_ = 0.9, $\gamma_{v}^{\prime }=\gamma_{d}^{\prime }=2.5$	β = 0.06, *w*_1_ = *w*_2_ = 0.3, $\gamma_{v}^{\prime }=\gamma_{d}^{\prime }=1.0$	β = 0.05, *w*_1_ = *w*_2_ = 0.7, $\gamma_{v}^{\prime }=\gamma_{d}^{\prime }=2.5$
VDA-RWR	*r* = 0.7, μ = 0.9, α = 0.5	*r* = 0.5, μ = 0.9, α = 0.9	*r* = 0.7, μ = 0.9, α = 0.9
VDA-KLMF	*r* = 6, *c* = 10, α = 0.1, λ_*A*_ = 1, λ_*B*_ = 4, *K* = 7	*r* = 45, *c* = 8, α = 0.2, λ_*A*_ = 3, λ_*B*_ = 2, *K* = 10	*r* = 35, *c* = 10, α = 0.8, λ_*A*_ = 6, λ_*B*_ = 5, *K* = 9

Recall (sensitivity), specificity, precision, F1 score, AUC, and AUPR are used to assess the performance of six VDA prediction approaches (VDA-KLMF, NGRHMDA, LRLSHMDA, NRLMF, VDA-KATZ, and VDA-RWR). Recall (sensitivity) indicates the ratio of correctly predicted positive VDAs to all known positive VDAs. Precision represents the ratio of correctly predicted VDAs to all predicted positive VDAs. Specificity denotes the ratio of correctly predicted negative VDAs to all known negative VDAs. F1 Score is the harmonic mean of recall and precision. The four evaluation metrics are defined as follows:


(21) $$Recall=\frac{TP}{TP+FN}$$

(22) $$Specificity=\frac{TN}{TN+FP}$$

(23) $$Precision=\frac{TP}{TP+FP}$$

(24) $$F1score=\frac{2TP}{2TP+FP+FN}$$

where *TP*, *FP*, *TN* and *FN* denote true positive, false positive, true negative and false negative, respectively. AUC is the average area under the Receiver Operating Characteristics (ROC) curve. The ROC curve is the plot of true positive ratio as a function of false positive ratio when the threshold to capture VDAs from the ranking varies. AUPR is the area under the Precision-Recall (PR) curve. The PR curve is the plot of true positive ratios among all predicted positive VDAs for each given recall value. AUPR provides a quantitative measurement of how well, on average, inferred association probabilities of positive VDAs are separated from the probabilities of negative VDAs. Higher recall, specificity, precision, F1 score, AUC and AUPR illustrate better performance. AUC and AUPR are two more important evaluation criterions compared to other four metrics.

### Performance Evaluation Under Three Five-Fold Cross Validations

VDA-KLMF is compared with NGRHMDA (Huang et al., 2017), LRLSHMDA (Wang et al., 2017), NRLMF (Liu et al., 2016), VDA-KATZ (Zhou et al., 2020), and VDA-RWR (Peng et al., 2021). The former three methods are representative association prediction approaches. NGRHMDA fused collaborative filtering and graph-based scoring. LRLSHMDA utilized a Laplacian regularized least square classifier. NRLMF used a neighborhood regularized Logistic matrix factorization model. The remaining two methods are state-of-the-art VDA prediction models. The two methods used the KATZ measurement and random walk with restart to prioritize anti-SARS-CoV-2 drugs, respectively. The experiments are repeated for 20 times and the final performance is averaged for 20 times. The results are shown in Tables 3–5. The best performance obtained from the six VDA prediction methods in each dataset is denoted in bold in each column.

**TABLE 3:** The performance of six VDA prediction methods on three datasets under CV1.

Datasets	Methods	Recall	Specificity	Precision	F1 score	AUC	AUPR
Dataset 1	NGRHMDA	**0.7278 ± 0.0411**	0.3997 ± 0.0071	0.0366 ± 0.0024	0.0643 ± 0.0039	0.7026 ± 0.0411	**0.4048 ± 0.0407**
	LRLSHMDA	0.1299 ± 0.0272	0.6170 ± 0.0034	0.0047 ± 0.0005	0.0084 ± 0.0009	0.1844 ± 0.0307	0.0121 ± 0.0001
	NRLMF	0.4933 ± 0.0072	0.6494 ± 0.0248	0.1572 ± 0.0168	0.1842 ± 0.0159	0.6621 ± 0.0260	0.1827 ± 0.0180
	VDA-KATZ	0.2616 ± 0.0499	0.5407 ± 0.0125	0.0125 ± 0.0015	0.0184 ± 0.0023	0.2683 ± 0.0543	0.0248 ± 0.0023
	VDA-RWR	0.4977 ± 0.0132	**0.7863 ± 0.0127**	0.0830 ± 0.0146	0.1055 ± 0.0111	**0.8157 ± 0.0130**	0.1090 ± 0.0266
	VDA-KLMF	0.6460 ± 0.0702	0.5122 ± 0.0081	**0.1640 ± 0.0228**	**0.2139 ± 0.0273**	0.7495 ± 0.0575	0.2538 ± 0.0598
Dataset 2	NGRHMDA	0.3987 ± 0.0107	0.5823 ± 0.0085	0.0461 ± 0.0007	0.0329 ± 0.0011	0.4301 ± 0.0098	0.0236 ± 0.0040
	LRLSHMDA	0.3507 ± 0.0077	0.4585 ± 0.0047	0.0435 ± 0.0001	0.0179 ± 0.0003	0.3173 ± 0.0053	0.0122 ± 0.0001
	NRLMF	0.5156 ± 0.0023	0.6303 ± 0.0134	0.1541 ± 0.0086	0.1895 ± 0.0078	0.6545 ± 0.0100	0.1614 ± 0.0094
	VDA-KATZ	0.5912 ± 0.0080	0.3143 ± 0.0039	0.0122 ± 0.0002	0.0232 ± 0.0003	0.3981 ± 0.0073	0.0142 ± 0.0001
	VDA-RWR	0.5106 ± 0.0025	**0.6840 ± 0.0079**	0.0620 ± 0.0025	0.0844 ± 0.0021	0.6932 ± 0.0074	0.0658 ± 0.0030
	VDA-KLMF	**0.7872 ± 0.0167**	0.5279 ± 0.0018	**0.1953 ± 0.0067**	**0.2618 ± 0.0089**	**0.8149 ± 0.0181**	**0.3487 ± 0.0224**
Dataset 3	NGRHMDA	0.4435 ± 0.0207	0.4699 ± 0.0122	0.0124 ± 0.0009	0.0232 ± 0.0017	0.4058 ± 0.0228	0.0817 ± 0.0158
	LRLSHMDA	0.1801 ± 0.0099	0.5777 ± 0.0021	0.0017 ± 0.0001	0.0074 ± 0.0003	0.2920 ± 0.0100	0.0077 ± 0.0001
	NRLMF	0.5416 ± 0.0056	**0.7266 ± 0.0201**	**0.1931 ± 0.0132**	**0.2086 ± 0.0119**	0.7591 ± 0.0146	0.2279 ± 0.0174
	VDA-KATZ	0.5712 ± 0.0185	0.3631 ± 0.0025	0.0117 ± 0.0005	0.0216 ± 0.0010	0.4639 ± 0.0173	0.0131 ± 0.0005
	VDA-RWR	0.5270 ± 0.0057	0.7021 ± 0.0115	0.0355 ± 0.0076	0.0812 ± 0.0071	0.7276 ± 0.0118	0.0372 ± 0.0092
	VDA-KLMF	**0.8040 ± 0.0373**	0.5179 ± 0.0029	0.1459 ± 0.0127	0.2044 ± 0.0172	**0.8224 ± 0.0406**	**0.3431 ± 0.0499**

*The best results are denoted in bold in each column.*

Table 3 lists the performance of six VDA identification models under CV1. It can be observed that VDA-KLMF computes the best recall, AUC, and AUPR, significantly outperforming NGRHMDA, LRLSHMDA, NRLMF, VDA-KATZ, and VDA-RWR on datasets 2 and 3. On dataset 1, VDA-KLMF calculates slightly lower recall, specificity, AUC, and AUPR than NGRHMDA and VDA-RWR. However, on dataset 2 and 3, VDA-KLMF obtains much better performance than the two approaches. It may be resulted in by small sample feature of dataset 1. The results demonstrate that abundant data can boost the prediction performance of VDA inference algorithms.

More importantly, the performance achieved by six VDA prediction models under CV1 is relatively lower than those of CV2 and CV3. The reason may be that there is a completely unknown virus in the three datasets, SARS-CoV-2, which does not show any associated drugs and thus decreases the prediction ability of these algorithms. Under the situation that few of any unlabeled drug for a new virus exists, VDA-KLMF can calculate the best AUCs of 0.8149 and 0.8224 and the best AUPRs of 0.3487 and 0.3431 on datasets 2 and 3, respectively. The result suggests that VDA-KLMF can be effectively applied to prioritize potential small molecules for a new virus, especially SARS-CoV-2.

Table 4 gives the performance of six VDA identification algorithms on the three VDA datasets under CV2. VDA-KLMF computes the best recall, F1 score, AUC and AUPR on all three datasets, much better than other five VDA techniques. For example, AUCs computed by VDA-KLMF are better 13.18, 13.98, 12.38, 12.28, and 4.65% than NGRHMDA, LRLSHMDA, NRLMF, VDA-KATZ, and VDA-RWR on dataset 1, respectively. It is better 7.23, 14.06, 8.92, 18.54, and 7.15% on dataset 2 and 24.13, 17.17, 13.38, 23.45, and 10.17% on dataset 3. AUPRs achieved from VDA-KLMF outperform 72.54, 48.01, 24.18, 40.32, and 63.52% compared to NGRHMDA, LRLSHMDA, NRLMF, VDA-KATZ, and VDA-RWR on dataset 1, respectively. Its performance outperforms 46.07, 41.00, 22.58, 40.88, and 47.14 on dataset 2 and 49.03, 46.31, 22.65, 42.90, and 42.52% on dataset 3. The comparative results demonstrate the superior prediction ability of VDA-KLMF for identifying possible viruses associated with a new drug.

**TABLE 4:** The performance of six VDA prediction methods on three datasets under CV2.

Datasets	Methods	Recall	Specificity	Precision	F1 score	AUC	AUPR
Dataset 1	NGRHMDA	0.6435 ± 0.0185	0.6713 ± 0.0112	0.0468 ± 0.0012	0.0850 ± 0.0021	0.8329 ± 0.0031	0.0674 ± 0.0074
	LRLSHMDA	0.7938 ± 0.0069	0.5762 ± 0.0049	0.0695 ± 0.0014	0.1122 ± 0.0014	0.8249 ± 0.0064	0.3127 ± 0.0240
	NRLMF	0.6069 ± 0.0085	0.7454 ± 0.0165	**0.4052 ± 0.0125**	0.3648 ± 0.0105	0.8409 ± 0.0106	0.5510 ± 0.0214
	VDA-KATZ	0.6889 ± 0.0120	0.6348 ± 0.0162	0.0925 ± 0.0168	0.1328 ± 0.0170	0.8419 ± 0.0096	0.3896 ± 0.0140
	VDA-RWR	0.5070 ± 0.0094	**0.8935 ± 0.0027**	0.1393 ± 0.0052	0.1294 ± 0.0047	0.9182 ± 0.0023	0.1576 ± 0.0062
	VDA-KLMF	**0.9148 ± 0.0078**	0.5459 ± 0.0028	0.3088 ± 0.0057	**0.3801 ± 0.0056**	**0.9647 ± 0.0086**	**0.7928 ± 0.0375**
Dataset 2	NGRHMDA	0.4867 ± 0.0116	0.8504 ± 0.0022	0.0395 ± 0.0005	0.0719 ± 0.0008	0.8017 ± 0.0008	0.0567 ± 0.0020
	LRLSHMDA	0.7720 ± 0.0036	0.4152 ± 0.0034	0.0085 ± 0.0007	0.0639 ± 0.0012	0.7334 ± 0.0029	0.1074 ± 0.0058
	NRLMF	0.5477 ± 0.0026	0.7476 ± 0.0094	**0.2669 ± 0.0062**	0.2787 ± 0.0046	0.7848 ± 0.0061	0.2916 ± 0.0079
	VDA-KATZ	0.5913 ± 0.0082	0.5699 ± 0.0107	0.0427 ± 0.0005	0.0696 ± 0.0004	0.6886 ± 0.0033	0.1086 ± 0.0052
	VDA-RWR	0.5045 ± 0.0020	**0.7982 ± 0.0029**	0.0454 ± 0.0007	0.0814 ± 0.0010	0.8025 ± 0.0029	0.0460 ± 0.0007
	VDA-KLMF	**0.8413 ± 0.0063**	0.5327 ± 0.0007	0.2309 ± 0.0041	**0.3003 ± 0.0038**	**0.8740 ± 0.0069**	**0.5174 ± 0.0241**
Dataset 3	NGRHMDA	0.4579 ± 0.0155	0.7070 ± 0.0042	0.0227 ± 0.0003	0.0279 ± 0.0007	0.6772 ± 0.0024	0.0351 ± 0.0015
	LRLSHMDA	0.7420 ± 0.0063	0.5235 ± 0.0020	0.0241 ± 0.0002	0.0493 ± 0.0005	0.7468 ± 0.0054	0.0623 ± 0.0067
	NRLMF	0.5592 ± 0.0056	**0.7424 ± 0.0114**	**0.2449 ± 0.0079**	0.2390 ± 0.0059	0.7847 ± 0.0075	0.2989 ± 0.0130
	VDA-KATZ	0.7246 ± 0.0068	0.3995 ± 0.0058	0.0297 ± 0.0002	0.0491 ± 0.0004	0.6840 ± 0.0058	0.0964 ± 0.0034
	VDA-RWR	0.5054 ± 0.0082	0.8087 ± 0.0064	0.0815 ± 0.0013	0.0628 ± 0.0019	0.8168 ± 0.0048	0.1002 ± 0.0013
	VDA-KLMF	**0.8935 ± 0.0112**	0.5245 ± 0.0011	0.1810 ± 0.0035	**0.2511 ± 0.0046**	**0.9185 ± 0.0119**	**0.5254 ± 0.0261**

*The best results are denoted in bold in each column.*

Table 5 shows recall, specificity, precision, F1 score, AUC, AUPR computed by six VDA prediction models on the three datasets under CV3. It can be seen that VDA-KLMF still obtains the best performance in terms of recall and AUC on the three datasets. Under CV3, NRLMF computes the best precision and F1 score on all datasets and is the second-best method. In particular, compared to NRLMF, recall obtained by VDA-KLMF is better 24.42, 26.90, and 27.41% on datasets 1–3, respectively. AUCs calculated by VDA-KLMF are better 6.14, 4.22, and 2.89%, respectively. AUPRs achieved from VDA-KLMF are better 11.20, and 4.31% on datasets 1–2, respectively. The results suggest that VDA-KLMF can effectively improve VDA prediction performance based on known VDAs.

**TABLE 5:** The performance of six VDA prediction methods on three datasets under CV3.

Datasets	Methods	Recall	Specificity	Precision	F1 score	AUC	AUPR
Dataset 1	NGRHMDA	0.5783 ± 0.0141	0.5582 ± 0.0160	0.0335 ± 0.0013	0.0615 ± 0.0024	0.6459 ± 0.0155	0.0410 ± 0.0035
	LRLSHMDA	0.8034 ± 0.0117	0.5804 ± 0.0050	0.0696 ± 0.0015	0.1119 ± 0.0017	0.8403 ± 0.0099	0.2838 ± 0.0212
	NRLMF	0.6482 ± 0.0073	0.7665 ± 0.0127	**0.4330 ± 0.0143**	**0.3961 ± 0.0120**	0.8679 ± 0.0092	0.6511 ± 0.0171
	VDA-KATZ	0.6976 ± 0.0118	0.6639 ± 0.0168	0.1067 ± 0.0101	0.1517 ± 0.0112	0.8803 ± 0.0106	0.3513 ± 0.0144
	VDA-RWR	0.4824 ± 0.0089	**0.8353 ± 0.0100**	0.1110 ± 0.0077	0.1153 ± 0.0058	0.8582 ± 0.0097	0.1268 ± 0.0100
	VDA-KLMF	**0.8924 ± 0.0094**	0.5440 ± 0.0008	0.3001 ± 0.0040	0.3670 ± 0.0055	**0.9392 ± 0.0103**	**0.7631 ± 0.0259**
Dataset 2	NGRHMDA	0.4544 ± 0.0053	0.3643 ± 0.0099	0.0112 ± 0.0002	0.0218 ± 0.0005	0.3011 ± 0.0055	0.0121 ± 0.0002
	LRLSHMDA	0.7838 ± 0.0050	0.4837 ± 0.0060	0.0757 ± 0.0008	0.0733 ± 0.0014	0.8248 ± 0.0020	0.0731 ± 0.0019
	NRLMF	0.5565 ± 0.0024	**0.7782 ± 0.0057**	**0.3000 ± 0.0046**	**0.3046 ± 0.0033**	0.8146 ± 0.0030	0.3335 ± 0.0062
	VDA-KATZ	0.5512 ± 0.0069	0.7558 ± 0.0124	0.0464 ± 0.0009	0.0805 ± 0.0013	0.8296 ± 0.0023	0.0834 ± 0.0028
	VDA-RWR	0.5022 ± 0.0016	0.6651 ± 0.0052	0.0326 ± 0.0008	0.0574 ± 0.0011	0.6675 ± 0.0049	0.0328 ± 0.0010
	VDA-KLMF	**0.8255 ± 0.0033**	0.5311 ± 0.0003	0.2077 ± 0.0016	0.2829 ± 0.0016	**0.8568 ± 0.0036**	**0.3766 ± 0.0076**
Dataset 3	NGRHMDA	0.3582 ± 0.0165	0.4423 ± 0.0208	0.0071 ± 0.0002	0.0119 ± 0.0005	0.2554 ± 0.0088	0.0078 ± 0.0006
	LRLSHMDA	0.8124 ± 0.0051	0.5237 ± 0.0023	0.0312 ± 0.0004	0.0552 ± 0.0008	0.8169 ± 0.0048	0.1057 ± 0.0103
	NRLMF	0.5890 ± 0.0038	**0.8028 ± 0.0076**	**0.3391 ± 0.0071**	**0.3191 ± 0.0057**	0.8572 ± 0.0048	**0.4155 ± 0.0107**
	VDA-KATZ	0.7116 ± 0.0166	0.5564 ± 0.0302	0.0359 ± 0.0010	0.0626 ± 0.0015	0.8478 ± 0.0042	0.0847 ± 0.0034
	VDA-RWR	0.5053 ± 0.0031	0.7049 ± 0.0068	0.0369 ± 0.0025	0.0556 ± 0.0024	0.7123 ± 0.0067	0.0374 ± 0.0028
	VDA-KLMF	**0.8631 ± 0.0072**	0.5224 ± 0.0004	0.1631 ± 0.0023	0.2331 ± 0.0025	**0.8861 ± 0.0076**	0.3906 ± 0.0158

*The best results are denoted in bold in each column.*

Under CV1, NGRHMDA calculates AUCs of 0.7026, 0.4301, and 0.4058 on three datasets, respectively. Under CV2, it computes AUCs of 0.8329, 0.8017, and 0.6772, respectively. Under CV3, it calculates AUCs of 0.6459, 0.3011, and 0.2554, respectively. Under CV1 and CV3, NGRHMDA computes AUCs smaller than 0.5 on datasets 2 and 3. In contrast, if we re-draw the ROC curve, it will obtain AUCs larger than 0.5 on the two datasets under CV1 and CV3. However, its computed AUCs will be smaller than 0.5 under CV2. Similarly, LRLSHMDA and VDA-KATZ compute AUCs smaller than 0.5 on three datasets under CV1, and ones larger than 0.5 under CV2 and CV3. In contrast, if we re-graph the ROC curve, the two methods will compute AUCs larger than 0.5 under CV1 and ones smaller than 0.5 under CV2 and CV3. It may be caused by their poor generalization ability.

**FIGURE 2:**
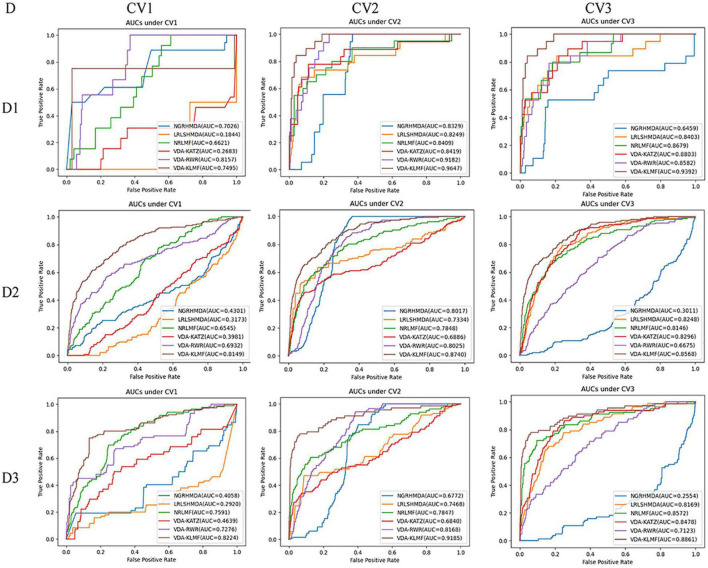
The AUC values predicted by six VDA prediction methods (D denotes dataset, Dl denotes dataset 1, D2 denotes dataset 2, D3 denotes dataset 3).

**FIGURE 3:**
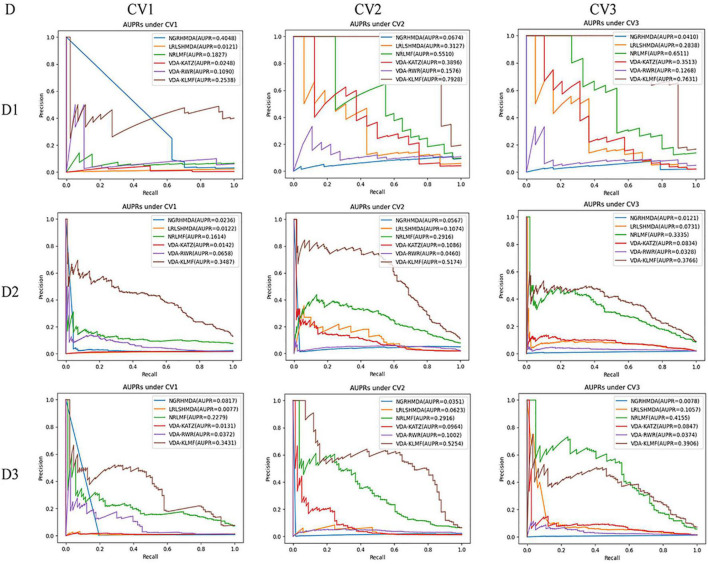
The AUPR values predicted by six VDA prediction methods (D denotes dataset, DI denotes dataset 1, D2 denotes dataset 2, D3 denotes dataset 3).

In addition, VDA-KLMF computes the slightly smaller specificity. However, specificity indicates the ratio of correctly predicted negative VDAs to all known negative VDAs. For anti-COVID-19 drug screening, it is possible anti-COVID-19 drugs that we need to capture. Therefore, it is more significant to find correctly predicted positive VDAs than correctly predicted negative VDAs. That is, sensitivity (recall) and precision are much more important than specificity. More importantly, under majority of situations, VDA-KLMF computes better AUCs and AUPRs, demonstrating relatively strong VDA prediction performance of VDA-KLMF. Figures 2, 3 depict the AUC and AUPR values calculated by six VDA prediction algorithms on three datasets under three different CVs, respectively.

### Effect of Gaussian Kernel on Virus-Drug Association Prediction Performance

**FIGURE 4:**
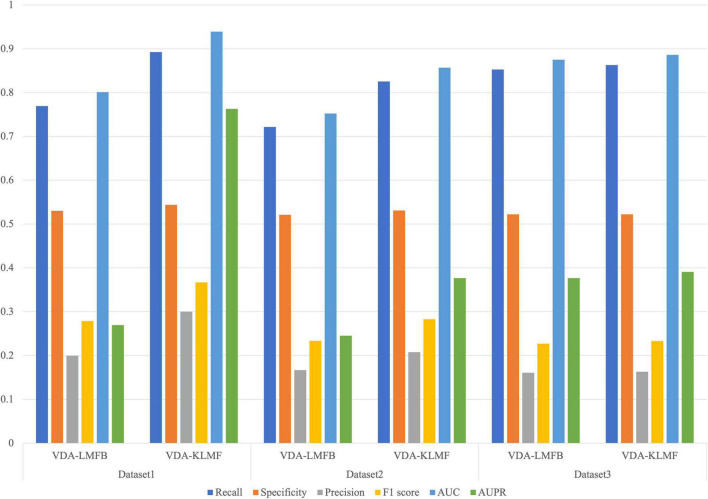
Effect of Gaussian kernel on virus-drug association prediction performance.

In the VDA-KLMF model, logistic matrix factorization model with kernel diffusion integrates Gaussian kernel and biological similarity kernel including sequence similarity of viruses and chemical structure similarity of drugs. Gaussian kernel fully utilizes the nearest neighbor information of viruses and drugs. We investigated VDA prediction performance of logistic matrix factorization model considering kernel diffusion with Gaussian kernel and biological similarity kernel (VDA-KLMF) and only considering biological similarity (VDA-LMFB). The results are shown in Figure 4. From Figure 4, we can find that kernel diffusion contributes to improving VDA identification ability.

### Effect of Different r-Values on the Performance of Virus-Drug Associations-Logistic Matrix Factorization Model

In the VDA-KLMF model, viruses and drugs are randomly mapped into two latent vector spaces AϵR^m×r^ and BϵR^n×r^ with the dimension of *r*. To evaluate the effect of different *r*-values on the prediction performance, we compared the performance of VDA-KLMF under different settings. Table 6 illustrates the comparison results of VDA-KLMF on three datasets under CV3. On dataset 1, we set *r* in the range of [2, 30] with the interval of 1. The results show that VDA-KLMF obtains the best prediction ability when *r* is set to 6. On datasets 2 and 3, we set *r* in the range of [5, 100] with the interval of 5. The results suggest that VDA-KLMF computes the best performance when *r* is set to 45 and 35, respectively. Therefore, the dimension *r* is set to 6, 45, and 35 on the three datasets, respectively.

**TABLE 6:** The effect of different *r* on VDA-KLMF on three datasets under CV3.

Datasets	Methods	Recall	Precision	F1 score	AUC	AUPR
Dataset 1	*r* = 2	0.8668 ± 0.0079	0.2794 ± 0.0034	0.3471 ± 0.0044	0.9105 ± 0.0088	0.6174 ± 0.0222
	*r* = 3	0.8820 ± 0.0098	0.2942 ± 0.0040	0.3614 ± 0.0043	0.9276 ± 0.0109	0.7205 ± 0.0258
	*r* = 4	0.8869 ± 0.0096	0.2986 ± 0.0043	0.3657 ± 0.0051	0.9331 ± 0.0106	0.7506 ± 0.0255
	*r* = 5	0.8896 ± 0.0056	0.2994 ± 0.0036	0.3661 ± 0.0046	0.9360 ± 0.0062	0.7614 ± 0.0209
	*r* = 6	**0.8924 ± 0.0094**	**0.3001 ± 0.0040**	**0.3670 ± 0.0055**	**0.9392 ± 0.0103**	0.7631 ± 0.0259
	*r* = 7	0.8887 ± 0.0121	0.2994 ± 0.0055	0.3659 ± 0.0070	0.9349 ± 0.0134	**0.7654 ± 0.0292**
	*r* = 8	0.8874 ± 0.0100	0.2997 ± 0.0033	0.3658 ± 0.0043	0.9339 ± 0.0109	0.7650 ± 0.0209
	*r* = 9	0.8895 ± 0.0111	0.2997 ± 0.0042	0.3659 ± 0.0056	0.9362 ± 0.0123	0.7568 ± 0.0208
	*r* = 10	0.8845 ± 0.0139	0.2986 ± 0.0051	0.3649 ± 0.0071	0.9304 ± 0.0150	0.7600 ± 0.0275
	*r* = 30	0.8839 ± 0.0086	0.2990 ± 0.0045	0.3653 ± 0.0052	0.9300 ± 0.0096	0.7592 ± 0.0243
Dataset 2	*r* = 5	0.8069 ± 0.0040	0.1965 ± 0.0026	0.2716 ± 0.0023	0.8363 ± 0.0044	0.3269 ± 0.0110
	*r* = 10	0.8195 ± 0.0030	0.2042 ± 0.0018	0.2791 ± 0.0016	0.8502 ± 0.0033	0.3619 ± 0.0089
	*r* = 15	0.8245 ± 0.0042	0.2070 ± 0.0021	0.2821 ± 0.0024	0.8556 ± 0.0046	0.3742 ± 0.0093
	*r* = 20	0.8241 ± 0.0030	0.2067 ± 0.0018	0.2819 ± 0.0019	0.8553 ± 0.0033	0.3729 ± 0.0089
	*r* = 25	0.8254 ± 0.0025	0.2072 ± 0.0014	0.2825 ± 0.0013	0.8567 ± 0.0004	0.3741 ± 0.0074
	*r* = 30	0.8250 ± 0.0038	0.2074 ± 0.0016	0.2824 ± 0.0019	0.8562 ± 0.0042	0.3761 ± 0.0073
	*r* = 35	0.8255 ± 0.0033	0.2070 ± 0.0017	0.2824 ± 0.0019	0.8568 ± 0.0035	0.3733 ± 0.0078
	*r* = 40	0.8250 ± 0.0032	0.2071 ± 0.0018	0.2824 ± 0.0016	0.8562 ± 0.0035	0.3748 ± 0.0083
	*r* = 45	0.8255 ± 0.0033	**0.2077 ± 0.0016**	**0.2829 ± 0.0016**	0.8568 ± 0.0036	**0.3766 ± 0.0076**
	*r* = 50	0.8241 ± 0.0036	0.2063 ± 0.0021	0.2816 ± 0.0019	0.8552 ± 0.0040	0.3709 ± 0.0097
	*r* = 100	**0.8262 ± 0.0041**	0.2074 ± 0.0024	0.2828 ± 0.0023	**0.8575 ± 0.0045**	0.3752 ± 0.0125
Dataset 3	*r* = 5	0.8401 ± 0.0065	0.1581 ± 0.0026	0.2252 ± 0.0027	0.8617 ± 0.0070	0.3620 ± 0.0184
	*r* = 10	0.8458 ± 0.0081	0.1536 ± 0.0026	0.2244 ± 0.0023	0.8677 ± 0.0086	0.3276 ± 0.0161
	*r* = 15	0.8458 ± 0.0081	0.1536 ± 0.0026	0.2244 ± 0.0023	0.8677 ± 0.0086	0.3276 ± 0.0161
	*r* = 20	0.8577 ± 0.0083	0.1603 ± 0.0032	0.2298 ± 0.0034	0.8804 ± 0.0088	0.3695 ± 0.0208
	*r* = 25	**0.8636 ± 0.0061**	0.1625 ± 0.0019	0.2326 ± 0.0021	**0.8866 ± 0.0065**	0.3836 ± 0.0131
	*r* = 30	0.8583 ± 0.0089	0.1597 ± 0.0036	0.2296 ± 0.0039	0.8810 ± 0.0095	0.3657 ± 0.0235
	*r* = 35	0.8631 ± 0.0072	**0.1631 ± 0.0023**	**0.2331 ± 0.0025**	0.8861 ± 0.0076	**0.3906 ± 0.0158**
	*r* = 40	0.8574 ± 0.0078	0.1608 ± 0.0031	0.2306 ± 0.0029	0.8800 ± 0.0083	0.3757 ± 0.0211
	*r* = 45	0.8622 ± 0.0047	0.1609 ± 0.0024	0.2312 ± 0.0020	0.8851 ± 0.0050	0.3710 ± 0.0182
	*r* = 50	0.8567 ± 0.0064	0.1554 ± 0.0024	0.2273 ± 0.0023	0.8791 ± 0.0068	0.3351 ± 0.0149
	*r* = 100	0.8585 ± 0.0048	0.1531 ± 0.0026	0.2238 ± 0.0023	0.8757 ± 0.0050	0.3199 ± 0.0222

*The best results are denoted in bold in each column.*

### Case Study

We wanted to identify potential chemical agents for preventing COVID-19 after confirming the powerful prediction ability of VDA-KLMF. We prioritized the top 10 compounds associated with SARS-CoV-2 on the three datasets. The results are shown in Tables 7–9, respectively. Among the top 10 small molecules with the highest association rankings with SARS-CoV-2, the majority of anti-SARS-CoV-2 drugs have been validated by current literatures. The results in Tables 7–9 show that there are seven available anti-SARS-CoV-2 compounds coming together on any two datasets, that is, remdesivir, ribavirin, nitazoxanide, favipiravir, emetine, chloroquine, and mycophenolic acid.

**TABLE 7:** The predicted top 10 drugs associated with SARS-CoV-2 on dataset 1.

Rank	Drug	Evidence
1	Remdesivir	PMID: 31996494, 32022370, 31971553, 32035018, 32035533, 32275812, 32145386, 32838064
2	Ribavirin	PMID: 32127666, 32227493
3	Oseltamivir	PMID: 32034637, 32127666
4	Zanamivir	PMID: 32294562
5	Mycophenolic acid	PMID: 32579258
6	Chloroquine	PMID: 32020029, 32145363, 32074550, 32236562
7	Peramivir	PMID: 32373347
8	Laninamivir	Unconfirmed
9	Rimantadine	PMID: 34344455
10	Presatovir	PMID: 33818470

**TABLE 8:** The predicted top 10 drugs associated with SARS-CoV-2 on dataset 2.

Rank	Drug	Evidence
1	Remdesivir	PMID: 31996494, 32022370, 31971553, 32035018, 32035533, 32275812, 32145386, 32838064
2	Emetine	PMID: 32251767
3	BCX4430 Galidesivir	PMID: 31389664
4	Niclosamide	PMID: 32361588, 33689873
5	Cyclosporine	PMID: 32505466, 32243698
6	Silvestrol	DOI: 10.1111/jcmm.15360
7	Mycophenolic acid	PMID: 32579258
8	Favipiravir	PMID: 32346491, 32967849, PMID: 32972430
9	Nitazoxanide	PMID: 32127666, 32568620, 32448490
10	Navitoclax	PMID: 33737523

**TABLE 9:** The predicted top 10 drugs associated with SARS-CoV-2 on dataset 3.

Rank	Drug	Evidence
1	Nitazoxanide	PMID: 32127666, 32568620, PMID: 32448490
2	Ribavirin	PMID: 32127666, 32227493
3	Chloroquine	PMID: 32020029, 32145363, 32074550, 32236562
4	Umifenovir	DOI: 10.2174/092986732766620041613111
5	Camostat	PMID: 32347443
6	Favipiravir	PMID: 32350860, 32967849, 33521757
7	Emetine	PMID: 32251767
8	Amantadine	PMID: 32361028
9	Hexachlorophene	PMID: 32366720
10	Irbesartan	Unconfirmed

Remdesivir is an adenosine triphosphate analogue. It has broad-spectrum antiviral activity and thus can be applied to the treatment of various diseases resulted in by the Arenaviridae, Flaviviridae, Filoviridae, Paramyxoviridae, Pneumoviridae, and Coronaviridae viral families (Malin et al., 2020). Remdesivir’s action against the Coronaviridae family makes it as a potential therapeutic strategy for COVID-19 (Gordon et al., 2020). On 19 November 2020, the drug in combination with baricitinib has been authorized to the treatment of COVID-19 (Eastman et al., 2020; FDA, 2021).

Ribavirin is a synthetic guanosine nucleoside (American et al., 2017). The small molecule can generate broad activity against a few RNA and DNA viruses by inhibiting the synthesis of viral mRNAs. It is widely applied to the treatment of hepatitis C and viral hemorrhagic fevers and might be effective in the early steps of viral hemorrhagic fevers (Myers et al., 2015; Wishart et al., 2018).

Nitazoxanide is a broad anti-infective compound. The drug can markedly modulate the survival, growth, and proliferation of various intracellular and extracellular protozoa, helminths, viruses, anaerobic and microaerophilic bacteria (Shakya et al., 2017). It can inhibit the replication of a few RNA and DNA viruses and has been investigated as a broad antiviral compound (Wishart et al., 2018).

### Molecular Docking

We conducted molecular dockings for the predicted antiviral drugs and the junction of the S protein-ACE2 interface. The binding energies between the predicted top 10 antiviral drugs on three datasets and the junction are shown in Table 10. From Table 10, we can observe that the identified top small molecules show higher binding energies with the junction, where nitazoxanide, mycophenolic acid, and zanamivir have the highest binding abilities. In addition, the key residues between the predicted seven compounds coming together on any two datasets and the junction are K68 and Q493 for remdesivir, R403, Q493, K353, and G496 for ribavirin, Q493 and S494 for nitazoxanide, K353 and G496 for favipiravir, T500 for emetine, H34 for chloroquine, and H34, K353, F390, and G496 for mycophenolic acid, respectively. The results suggest that K353 and G496 are possible key residues between anti-SARS-CoV-2 drugs and the junction of the S protein-ACE2 interface.

**TABLE 10:** Binding energy between the predicted antiviral drugs and the junction of the S protein-ACE2 interface.

Drug	Binding energy (kcal/mol)	Drug	Binding energy (kcal/mol)
Remdesivir	–7.00	BCX4430 Galidesivir	–6.87
Ribavirin	–6.59	Camostat	–7.48
Nitazoxanide	–7.74	Cyclosporine	–8.92
Favipiravir	–5.32	Hexachlorophene	–7.67
Emetine	–6.95	Irbesartan	–8.13
Chloroquine	–5.82	Laninamivir	–5.7
Mycophenolic acid	–7.0	Navitoclax	–8.39
Rimantadine	–6.63	Niclosamide	–8.06
Silvestrol	–5.54	Oseltamivir	–6.5
Umifenovir	–6.89	Peramivir	–6.88
Zanamivir	–5.96	Presatovir	–8.38

**FIGURE 5:**
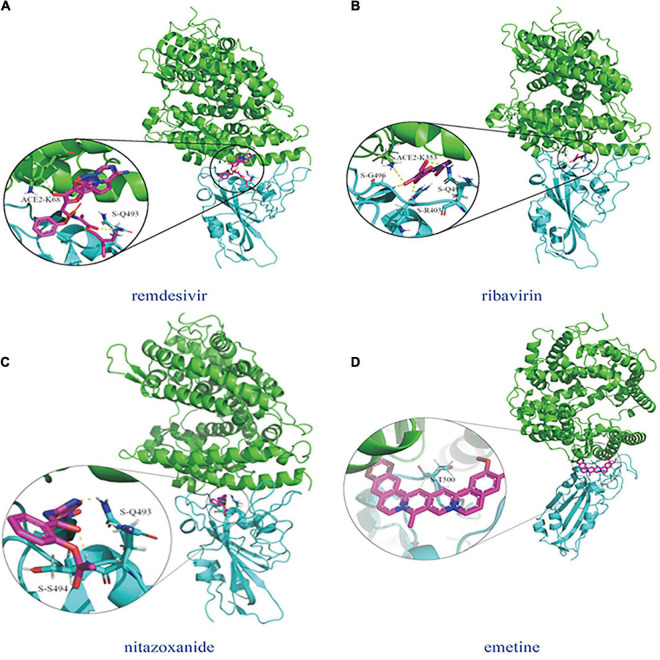
Molecular dockings between the predicted four possible antiviral drugs against COV1D-19 (remdesivir, ribavinn, nitazoxanide, and emetine) and the junction of the S protein-ACE2 interface. **(A)** remdesivir (Peng et al., 2021; Wang J. et al., 2021; Shen et al., 2022), **(B)** ribavirin (Peng et al., 2021; Wang J. et al., 2021; Shen et al., 2022), **(C)** nitazoxanide, and **(D)** emetine.

Molecular dockings between the predicted four possible antiviral drugs against COVID-19 (remdesivir, ribavirin, nitazoxanide, and emetine) and the junction are illustrated in Figure 5, where two docking graphs [(a) remdesivir and (b) ribavirin] were provided by Peng et al. (2020). The subfigure in each circle denotes the residues at the junction and their corresponding orientations. Green denotes the structure of ACE2 and cyan denotes the SARS-CoV-2 S protein.

## Discussion

Since the outbreak of COVID-19, we conducted several works for initially screening possible drugs applied to this highly contagious disease based on virus sequences, drug chemical structures, and observed VDAs from existing data resources. These works include VDA-RLSBN (Peng et al., 2020), VDA-RWR (Peng et al., 2021), and the proposed VDA-KLMF methods. VDA-RLSBN and VDA-RWR first utilized complete genomic sequences of viruses and chemical structures of drugs. Second, they developed computational models to detect underlying associations between SARS-CoV-2 and small molecules. Finally, they conducted molecular dockings between the predicted anti-COVID-19 drugs and two target proteins including the S protein and ACE2 to measure their binding ability. The two methods effectively captured possible antiviral drugs against COVID-19.

In particular, VDA-KLMF integrates drug chemical structures, virus sequences, known VDAs, Gaussian kernel, similarity kernel, and logistic matrix factorization with kernel diffusion. It is compared with two state-of-the-art VDA prediction models and three classical association inference methods. The experimental results illustrate that the proposed VDA-KLMF method obtains powerful prediction performance.

SARS-CoV-2 is a new virus, that is, an orphan node in a VDA network. It has no association with available drugs. To capture underlying FDA-approved drugs against SARS-CoV-2, VDA-KLMF computes sequence similarity between the virus and other viruses and obtains a similarity matrix with the elements in the range of (0,1). Based on sequence similarity kernel and Gaussian kernel, VDA-KLMF can predict association information for SARS-CoV-2 combining matrix factorization model with kernel diffusion. The results show that four small molecules, remdesivir, ribavirin, nitazoxanide, and emetine, have higher binding energies with the junction of the S protein-ACE2 interface.

VDA-KLMF computes superior prediction performance. It has the following three characteristics. First, it effectively integrates various biological information including global and local similarities of viruses and drugs. Second, logistic matrix factorization model with kernel diffusion more accurately quantifies the interplays between viruses and drugs. Finally, two key residues (K353 and G496) are found and need further medical validation.

Compared to VDA-KLMF, VDA-RLSBN remains the following four limitations: (i) Its prediction ability was only validated on one dataset comprised of 96 VDAs between 12 viruses and 78 drugs, which may possibly result in the overfitting problem. (ii) It was only evaluated under CV3 and failed to measure the performance under CVs on viruses and drugs, thereby failures to investigate its generalization ability. (iii) It found 10 potential small molecules against COVID-19 from 78 FDA-approved drugs on the constructed small dataset. Drugs that may be applied to screen the clues of treatment for patients with the infection of COVID-19 are relatively few ones. (iv) It implemented molecular dockings between the identified small molecules and the target proteins including the S protein and ACE2, respectively. In comparison, our proposed VDA-KLMF method use three datasets and is evaluated under CVs on viruses, drugs and VDAs. In this context, VDA-KLMF obtains better performance, thereby demonstrating its powerful generalization ability. Moreover, VDA-KLMF screens possible anti-COVID-19 drugs coming together in any two datasets and the inferred results may be more reliable than those from unique dataset. Finally, VDA-KLMF conducts molecular dockings between the screened drugs and the junction of the S protein-ACE2 interface, which can more reasonably measure their binding abilities.

Similar to VDA-KLMF, VDA-RWR was also measured under three CVs on three datasets. AUC and AUPR are two more important evaluation metrics compared to recall, precision, specificity, and F1 score. VDA-KLMF significantly outperforms VDA-RWR under the above situations. The results illustrate that VDA-KLMF can more precisely screen potential drugs against COVID-19, while further accurately prioritizing possible small molecules during the initial drug screening is vital to the treatment of COVID-19. More importantly, VDA-KLMF captures two candidate drugs (nitazoxanide and emetine) except remdesivir and ribavirin and provides more choices to initially screen available compounds against COVID-19.

To better uncover potential therapeutic clues for COVID-19 and similar diseases produced by evolving SARS-CoV-2, in the future, first, we will build a bigger and SARS-CoV-2-related database comprised of drugs, disease, and targets. Second, abundant biological data related to single strand RNA viruses should be integrated to more accurately depict biological features of viruses and drugs. Finally, a more robust model, for example, deep learning model, should be built to boost VDA identification performance. We anticipate that this work can contribute to the initial drug screening for therapy of patients with the infection of COVID-19.

## Data Availability Statement

The datasets presented in this study can be found in online repositories. The names of the repository/repositories and accession number(s) can be found in the article/Supplementary Material.

## Author Contributions

XT, LP, and LZ: conceptualization. XT and LP: methodology and writing—review and editing. XT and LH: software. XT, LS, PG, and GL: validation. LP and LZ: investigation, supervision, project administration, and funding acquisition. XT, LS, and GL: data curation. LP: writing—original draft preparation. LS: visualization. All authors have read and agreed to the published version of the manuscript.

## Conflict of Interest

The authors declare that the research was conducted in the absence of any commercial or financial relationships that could be construed as a potential conflict of interest.

## Publisher’s Note

All claims expressed in this article are solely those of the authors and do not necessarily represent those of their affiliated organizations, or those of the publisher, the editors and the reviewers. Any product that may be evaluated in this article, or claim that may be made by its manufacturer, is not guaranteed or endorsed by the publisher.
